# The predictive validity of the V-RISK-10 and BVC among involuntarily admitted patients

**DOI:** 10.3389/fpsyt.2024.1342445

**Published:** 2024-02-27

**Authors:** Tilmann Kös, Peter Bräunig, Joscha Hausam

**Affiliations:** ^1^ Vivantes Humboldt-Klinikum, Vivantes Netzwerk GmbH, Berlin, Germany; ^2^ Institut für Forensische Psychiatrie, Charité – Universitätsmedizin Berlin, Corporate Member of Freie Universität Berlin and Humboldt-Universität, Berlin, Germany

**Keywords:** risk assessment, violent behavior, acute psychiatry, mental disorder, involuntary admission

## Abstract

Although endangerment towards others is a criterion for an involuntary admission in many countries, research on risk assessment of endangerment among involuntarily admitted individuals is limited. In this retrospective case-control study, we calculated scores for a German-translated version of the Violence Risk Screening-10 (V-RISK-10) and the Brøset Violence Checklist (BVC) in a sample of 111 people undergoing an involuntary admission in Reinickendorf, Berlin. Outcomes were violence, coercive measures, and readmission. In line with our hypotheses, the BVC demonstrated stronger predictive validities for short-term, and V-RISK-10 for long-term events. There was an incremental validity for both instruments for restraint 24 hours after admission and any violence until discharge. These findings support the evidence that structured risk assessment instruments may be useful for individuals undergoing an involuntary admission. Ethical considerations about screening procedures are discussed.

## Introduction

In modern psychiatry, the purpose of risk assessment is to identify and prevent potential harmful events, such as suicidality or violence. Risk assessment strategies can be divided into unstructured professional judgement (UPJ), and structured methods, that is, actuarial risk assessment instruments (ARAI’s) and structured professional judgements (SPJ) (1). While UPJ reflects a personal intuition or opinion, structured methods use observable static and dynamic risk factors (2, 3). There is an emerging consensus that structured methods yield better results than UPJ (3, 4). However, research on violence risk assessment in general psychiatry is scarce. Most of previous research had been dedicated to forensic and prison psychiatry (5, 6), thus, the generalization for the general psychiatric setting is questionable. Moreover, existing risk instruments do not align with the fast-paced, high stress environment of general psychiatry since the majority is time-consuming and requires a specific training (7). For this purpose, screening procedures are required. To broaden the scope for general psychiatry, we examined the predictive validity of the Brøset Violence Checklist (BVC, 8) and the Violence Risk Screening-10 (V-RISK-10, 9). The focus in this study is on individuals who have been involuntarily admitted to an acute psychiatric unit due to endangerment.

In Germany, people can be involuntarily admitted to an acute psychiatric unit based on the civil right commitment by a legal guardianship (BGB, “Bürgerliches Gesetzbuch”) or the mental health laws (PsychKG, “Psychisch-Kranken-Gesetze”). According to the BGB, solely subacute self-endangerment, such as neglect or chronification, is a criterion for an involuntary admission. The PsychKG can be both applicated for acute self-endangerment and endangerment towards others, such as suicidality or severe violence, which will be the main scope of this study.

In view of the ethical tightrope walk between assessing violence risk and preserving the rights of the individuals, structured risk assessment could support the evaluation process for those undergoing an involuntary admission. From one perspective, there is some evidence that violence is highly prevalent in general psychiatry, especially among individuals being involuntarily admitted. For instance, one meta-analysis found that 17% of psychiatric patients are involved in at least one violent act (8). Additionally, a study focusing on individuals under involuntary admission revealed that 49.5% of the patients under civil commitment exhibited violent threats towards others (9). Conversely, involuntary admissions represent a significant disruption in individuals’ lives. As they can have detrimental effects (10, 11), including a higher risk of leading to further coercive measures (12, 13), an involuntary admission (i.e. the determination and duration) needs to be proportional to the burden that it may impose on the individual (14).

For the general psychiatric context, a small number of risk assessment instruments is recommended to apply (7). Risk assessment instruments for the general psychiatric field are commonly divided along the predicted time-period, that is, into short- (8h – 72h) and long-term risk assessment (weeks to years). It is important to note, that there is no clear definition at what time point short- and long-term instruments are divided. The BVC (short-term, 8) and V-RISK-10 (long-term, 9) have currently been recommended for further research (7). The BVC was developed to predict imminent violence up to 72 hours by rating behavioral changes, such as confusion, irritability and verbal threats (15). The V-RISK-10 is a screening tool to identify individuals with a possible risk from days to a year based on the evaluation of static and dynamic risk factors (16, 17).

While there is a growing body of research on the predictive validity of the BVC, there is, to date, relatively limited research on the predictive validity for the V-RISK-10. For the BVC, a recent meta-analysis revealed 62 studies with a robust predictive validity (AUC range between .69-.98, 18). To the best of our knowledge, ten studies have been conducted on the V-RISK-10 up to now. The V-RISK-10 was both investigated for the inpatient and outpatient context, leading to AUC values from .68-.80 regarding violence (16, 17). However, for people undergoing an involuntary admission, the predictive validity for any violence after discharge tends to be lower with an AUC of .69 (17). Further research is required to replicate previous findings for the V-RISK-10.

Additionally, several open questions surround the utilization of the V-RISK-10: Firstly, initial attempts have been made to translate the V-RISK-10 into various languages, including Persian and Chinese (18, 19). To date, there is no German-translated version available. Secondly, Anderson and Jenson (2019) recommended to investigate the incremental validity when used in conjunction with BVC (7). Nonetheless, a recent study examined the incremental validity solely using one item of the V-RISK-10 (20). In terms of generalizing the instrument towards specific subpopulations, an adapted version for young people exists (21). Consequently, adaptations for additional samples, such as involuntarily admitted patients, could broaden its practical utility. Lastly, there has been no study assessing the predictive validity for coercive measures, such as medication and restraint.

### Aims of our study

The aim of the present study was to examine the predictive validity of structured risk assessments for predicting short- and long-term violence in psychiatry. The first aim of our study was to investigate the predictive validity of a German version of the V-RISK-10. The second aim was to compare the predictive validity with the BVC related to short- and long-term outcome criteria, such as violence (non-physical, physical), coercive measures (restraint, medication) and readmissions (voluntary, involuntary), respectively. We defined different hypotheses regarding the duration of the outcome criteria (short-, long-term) as follows:

1. We hypothesized significant predictive validities between the BVC and short-term events occurring 24-72 hours after admission, such as violence and coercive measures.
2. We expected significant predictive validities between V-RISK-10 at admission with long-term events occurring in the time-period 72 hours after admission.
3. For events after discharge, we expected significant predictive validities for the V-RISK-10.
4. For the BVC sum score at admission, we did not expect a significant predictive validity for any event after discharge.
5. Further we hypothesized that the V-RISK-10 augments the predictive validity of the BVC.

## Methods

### Setting and sample

The study was conducted at the acute psychiatric unit in Vivantes-Humboldt-Klinikum (HUK), Berlin-Reinickendorf. It is a hospital with a mandate for healthcare provision, serving a population of approximately 270,000 people. Based on the mental health laws, people showing signs of a psychiatric disorder and who are in acute endangerment for others or themselves, the social psychiatric service (SPS) is obliged to order an involuntary admission. The admission process is twofold: Firstly, medical staff of the social psychiatric service (SPS) writes a report about the conditions and circumstances. Secondly, not later than the following day, an on-call judge needs to personally examine the patient. An involuntary admission is permitted by an on-call judge for up to six weeks.

The target population was adult individuals (≥ 18 years) who were admitted to the acute psychiatric unit within three years from the 01.01.2018 to the 31.12.2020 due to endangerment towards others. All reports by the SPS in this time-period (*N* = 254) have been analyzed regarding information about self-endangerment and endangerment towards others. Due to scan quality, *n* = 11 reports were not readable and were excluded from analyses. As both self-endangerment and endangerment towards others meet the requirements for an involuntary admission, in the present study we included patients who presented any form of endangerment towards other people according to the SPS (*n* = 123). Seven patients were re-referred at least one time during 2018 and 2020, so cases with additional readmissions were excluded to avoid confoundings (*n* = 12). The final sample comprises *n* = 111 patients.

**Table 1** illustrates the main characteristics of the sample. Sixty-two percent were males, a psychotic disorder was most diagnosed (43.2%). Every fifth person had a substance-associated co-diagnosis (21.6%). Regarding socio-demographic characteristics, the majority was unemployed (72.1%) and not in a relationship (83.8%). Every fifth person had no German nationality (21.9%), five percent were homeless. Concerning legal characteristics, every second person was voluntarily admitted before (50.5%). Regarding involuntary admissions, almost every third person was admitted via PsychKG (30.6%) and more than every fifth person via BGB (22.5%), respectively. However, almost half of the sample was under legal guardianship (47.4%). More than every third person visited the ambulant service in the clinic 12 months before admission (36.9%). The average recommended length of stay via the SPS was *M* = 26.7 days (*SD* = 11.8). The average length of stay, as determined by the judge, was *M* = 16.4 days (*SD* = 13.6). The average duration of hospitalization for all individuals was *M* = 34.8 days (*SD* = 41.9). Because the duration is biased by individuals undergoing a civil commitment under BGB, after excluding those individuals (*n* = 20) the average duration of hospitalization was *M* = 25.7 days (*SD* = 35.3).

**Table 1:** Sociodemographic and clinical characteristics of the sample at admission (*N* = 111).

Characteristics	*M (SD)*	% (*N*)
Age	48.4 (20.9)	
Sex		
Male		62.2 (69)
Female		37.8 (42)
Primary diagnosis		
F0		15.3 (17)
F1		14.4 (16)
F2		43.2 (48)
F3		1.8 (2)
F31		15.3 (17)
F4		5.4 (6)
F5		0 (0)
F6		0.9 (1)
F7		2.7 (3)
F8		0.9 (1)
F9		0
Alcohol or drug associated co-diagnosis		21.6 (24)
Relationship^a^		16.2 (18)
Unemployment^a^		72.1 (80)
Homelessness		5.4 (6)
German nationality		79.1 (88)
Previous stays		
Voluntary		50.5 (56)
Involuntary: PsychKG		30.6 (34)
Involuntary: BGB		22.5 (25)
Psychiatric institute ambulance in the last 12 months		36.9 (41)
Legal guardianship		47.4 (53)
Applied duration (days) of stay via SPS	26.7 (11.8)	
Determined duration of stay via court	16.4 (13.6)	
Duration of hospitalization (all cases)	34.8 (41.9)	
Duration of hospitalization (no BGB during hospitalization)^b^	25.7 (35.5)	
BVC at admission sum score	2.30 (1.80)	
V-RISK-10 at admission sum score	10.81 (4.90)	
V-RISK-10 at discharge sum score	8.41 (5.33)	

PsychKG, Psychisch-Kranken-Gesetze; BGB, Bürgerliches Gesetzbuch; SPS, Social Psychiatric Service; BVC, Brøset Violence Checklist; V-RISK-10, Violence Risk Screening-10.

^a^one missing. ^b^n = 91.

### Procedure

Sociodemographic data were collected by T. K. and psychology students based on a routine documentation called “Basisdokumentation” (BaDo-Bogen) that had been filled out by medical professionals during hospitalization. If information was not given, further information based on documents, such as medical reports and letters were researched and supplemented in the database.

The BVC was retrospectively applied by T. K. from the nurses and medicals first contact after admission. The V-RISK-10-Score was retrospectively applied by T. K. from the nurses and medicals first contact, the anamnesis and data from previous stays.

To achieve blinding in data collection, data on outcomes were collected by psychology students. For short-term outcome-criteria, data had been collected from the clinical digital system containing reports from the medical first responders and nurses originally taken 24 hours and 72 hours after admission.

### Measures

#### BVC

The BVC is a risk assessment screening tool to predict short term inpatient violent behavior within 24 hours after assessment (15). The evaluation procedure is based on the assessment of absence (= 0) and presence (= 1) of six items. The items reflect three patient characteristics (confusion, irritability and boisterousness) and three behavioral features (physical threats, verbal threats and attacking objects) which often occur in psychiatric patients before violent behavior (22). No specific qualification or training is required (15). Though a total score is typically divided into three risk categories (small, moderate, high risk), each reflecting a higher risk of violent behavior, different cut-off scores are reported [≥ 4 (20) and ≥ 3 for high risk (15)]. Longitudinal and randomized-control trials suggest sufficient reliability and validity for short-term violent behavior (15, 23, 24). In line with the objective of predicting short-term events, BVC items were solely rated at admission and not at discharge.

#### V-RISK-10

The V-RISK-10 has been developed for civil psychiatric patients to facilitate an immediate quick-check strategy in acute psychiatric settings (16). Compared to time-consuming risk assessment instruments from the forensic psychiatric field, it is supposed to require a screening method to exclude patients with low risk features (16). The V-RISK-10 comprises ten items divided into static (e.g. previous/current violence, previous/current major mental disorder, personality disorder) and dynamic risk factors (eg. lack of insight, suspicion, unrealistic planning) that are assessed by staff with medical-psychiatric experience. However, a specific training is not required (16, 21). The procedure comprises three steps including standardized and structural judgements. First, based on file reviews and clinical observations, each item needs to be evaluated on a 3-range scale (no = 0, maybe/moderate = 1, yes = 2). Numbers are summed up to a total value reflecting an actuarial procedure (ARAI). Second, a summarizing risk-evaluation into one out of three risk categorizations needs to be assessed (low, moderate, high), reflecting an SPJ. For practice, an evaluation of a further need of detailed risk-assessment or measures is required (none, further risk assessment, implementation of preventive measures), however, in this study we will focus on the standardized procedure (ARAI). Results from prospective studies show good reliability and validity scores (25). For 34 cases, there was at least one missing item. In line with the methodological steps reported in a previous study (16), we conducted a prorating procedure for missing items. According to the objective of predicting events after admission and discharge, V-RISK-10 items were both rated at admission and discharge.

### Translation procedure

The translation procedure was conducted according to the World Health Organization Guidelines (26). A bilingual psychologist translated the V-RISK-10 into German. The German version was then translated back into English by a second bilingual psychologist. Both bilingual translators are native speakers in both languages. Discrepancies between both English versions were discussed by an expert panel (T. K., medical staff of the clinic and one psychologist with 5 years experiences in a forensic institution). The draft version was tested for comprehensibility by three students (one woman and two men ages 20-30). To determine the interrater reliability, we conducted a pilot study with two assessors (T. K. and one psychologist with 5 years experiences in a forensic institution) and *N* = 30 cases in July 2021. Intraclass correlation coefficients revealed sufficient values for both the sum score (ICC = .83, 95% *CI* [.79;.86]) and the Structured Professional Judgement (ICC = .74, 95% *CI* [.56;.86]), respectively. ICC values were thus comparable to studies from other countries (18, 25, 27).

### Outcome criteria

Outcome criteria were violent behavior (physical and non-physical) and coercive measures (restraint and medication) at different time points (short-term = 24-72 hours, long-term = until and after discharge). Individuals who had been discharged during these time-periods were excluded from analyses (72 hours: *n* = 12, stay: *n* = 16). Outcome criteria were collected for time-points after discharge in line with previous studies (long-term = 3 months, 12 months, 2 years, 20). Additionally, involuntary and voluntary readmissions after discharge were collected. For the follow-up-period after discharge, two patients were excluded who died during their stay. Further, three patients died during the follow-up period, and thus have been excluded for the six month (*n* = 1), one year (*n* = 1) and two year follow-up (*n* = 1), respectively. For two patients, there was a sufficient follow-up time available, rendering exclusion insignificant.

Violent behavior was defined in line with past studies as a “physical act against another person involving the use of body parts or objects, with a clear intent to cause physical injury to that person, or verbal communications that convey a clear intent to inflict physical injury on another person.” (28). Furthermore, in line with previous studies (16), we categorized the variable “any violence” into “physical” and “non-physical violence” to distinguish between mild and severe forms of violence.

Coercive measures were operationalized as follows: For coercive medication, we coded any event where healthcare professionals attempt to persuade the patient to take medication or where a medication was administered during restraint. Due to the strict legal regulations in Germany regarding restraints, information was gathered from official documents that are routinely checked and documented by employees. Involuntary readmissions included solely admissions under PsychKG and not BGB, since BGB is exclusively for cases including self-endangerment.

### Statistical analyses

The predictive validity of the BVC and V-RISK-10 was investigated with Area under the curve (AUC) values. AUC values are based on Receiver Operating Characteristic Analysis (ROC, 29) where the true positive (sensitivity) and false positive (specificity) rates are plotted in a diagram. AUC values represent the area under the ROC curve itself, indicating a higher discrimination by a larger area (29). ROC analysis and AUC values are less sensitive to low base rates, providing a useful tool for rare events, such as violence or coercive measures. Differences between AUC values were calculated using the DeLong method (30). Differences were calculated exclusively for AUC values that demonstrated significant predictive validity. Effect sizes with a value of .56, .64, .71 represent a small, medium and large effect size, respectively (31). To compare cut-off points of previous studies of the BVC (15, 20) and V-RISK-10 (17, 18, 27) for violent behavior, we calculated optimal cut-off scores based on the Youden’s index (32). The Youden’s index is determined by maximizing the difference between the true positive and false positive rate.

Incremental validity was examined by stepwise binomial logistic regression analyses (33) with the BVC and V-RISK-10 as independent variables, and every outcome criterion as dependent variables. For each outcome criterion, we first calculated a regression analysis with the BVC. Secondly, we added the V-RISK-10 to the regression model. The overall model fit was determined by Nagelkerke-*R* ^2^. A significant change of the overall model fit after adding the V-RISK-10 was calculated via Likelihood-Ratio test (LL test), indicating incremental validity. There were no autocorrelations of the residuals (Durbin-Watson-statistics = 1.92-2.08, *p* = ns) and no multicollinearities between the predictors (V-RISK-10 and BVC: *r* = .55, *p* <.001). However, residuals between the predictors and the logit transformation of the dependent variable were not randomly distributed, indicating a violation of one assumption. Despite this, logistic regression analyses were calculated because of its practical relevance and robustness. For all tests, a significance level of 0.05 was selected. Data analyses were carried out with the statistical software R, Version 3.5.3 (34).

## Results

The single values for measures and instruments were as follows: The BVC total average score for the sample at admission was *M* = 2.30 (*SD* = 1.80) with an acceptable internal consistency (Cronbach’s α = .76). The V-RISK-10 total average score at admission for the sample was *M* = 10.81 (*SD* = 4.90) with an acceptable internal consistency (Cronbach’s α = .81). The V-RISK-10 average score at discharge was *M* = 8.41 (*SD* = 5.33). Moreover, there were high correlations between the BVC and the V-RISK-10 at admission (*r* = .55, *p* <.001).

### Predictive validity during admission

The predictive validity of the two structured risk assessment scores for the outcomes are reported in **Table 2**. Both BVC and V-RISK-10 at admission predicted short-term events occurring 24 and 72 hours after admission at a significant level, indicating large effects (all AUC ≥.73, *p* <.006). Additionally, for both instruments there were predictive validities for almost all events until discharge, indicating large effects (AUC ≥.74, *p* <.001).

**Table 2:** Predictive validity of BVC and V-RISK-10 for inpatient violence and coercive measures until discharge.

Variables			BVC			V-RISK-10			Differences between V-RISK-10 and BVC
	*n*	cases	AUC	*p*	95% *CI*	AUC	*p*	95% *CI*	*p*
24 hours after admission									
Any violent behavior	111	24	.94	<.001	.89, .98	.83	<.001	.75, .91	.013
Non-physical	111	23	.93	<.001	.88, .98	.81	<.001	.73, .89	.006
Physical	111	13	.95	<.001	.92, .99	.81	.002	.71, .91	.006
Any coercive measure	111	24	.91	<.001	.81, .96	.81	<.001	.72, .90	.019
Medication	111	23	.87	<.001	.80, .95	.80	<.001	.71, .80	.097
Restraint	111	21	.89	<.001	.81, .96	.83	<.001	.75, .91	.214
72 hours after admission									
Any violent behavior	99	30	.90	<.001	.82, .97	.79	<.001	.69, .88	.019
Non-physical	99	29	.89	<.001	.82, .97	.77	<.001	.68, .86	.012
Physical	99	20	.92	<.001	.86, .97	.73	.006	.62, .84	<.001
Any coercive measure	99	24	.92	<.001	.86, .97	.78	<.001	.68, .88	.025
Medication	99	24	.89	<.001	.82, .96	.76	<.001	.66, .86	.009
Restraint	99	21	.90	<.001	.83, .98	.81	<.001	.71, .91	.083
Until discharge									
Any violent behavior	95	36	.85	<.001	.77, .94	.79	<.001	.69, .88	.212
Non-physical	95	36	.85	<.001	.77, .94	.79	<.001	.69, .88	.212
Physical	95	25	.84	<.001	.75, .94	.74	.001	.65, .86	.128
Any coercive measure	95	30	.87	<.001	.80, .95	.80	<.001	.71, .90	.193
Medication	95	29	.86	<.001	.79, .94	.79	<.001	.70, .89	.168
Restraint	95	21	.88	<.001	.80, .96	.81	<.001	.70, .91	.187

BVC, Brøset Violence Checklist; V-RISK-10, Violence Risk Screening-10.

### Predictive validity after discharge

The predictive validities between the BVC and V-RISK-10 for post-discharge events are reported in **Table 3**. For the V-RISK-10 at discharge, there were predictive validities for each outcome with large effects (AUC ≥.71, *p* <.05). For the BVC, there were three significant predictive validities for any violence and non-physical violence three months after discharge (both AUC = .74, *p* <.01) and for non-physical violence one year after discharge (AUC = .69, *p* <.05).

**Table 3:** Predictive validity of BVC and V-RISK-10 for violence, coercive measures and readmissions at post-discharge.

Variables			BVC at admission (*n* = 109)			V-RISK-10 at discharge (*n* = 109)			Differences between V-RISK-10 and BVC
	*n*	cases	AUC	*p*	95% *CI*	AUC	*p*	95% *CI*	
Clinical outcomes									
3 months after discharge									
Any violent behavior	109	17	.74	.007	.60, .89	.94	<.001	.91, .99	.008
Non-physical	109	17	.74	.007	.60, .89	.81	<.001	.72, .90	.454
Physical	109	13	.68	.088	.50, .86	.79	.003	.68, .91	–
Any coercive measure	109	13	.64	.210	.45, .83	.82	.001	.76, .92	–
Medication	109	12	.64	.202	.44, .85	.86	<.001	.75, .98	–
Restraint	109	12	.69	.091	.51, .87	.86	<.001	.74, .98	–
1 year after discharge									
Any violent behavior	108	28	.63	.113	.51, .75	.87	<.001	.79, .95	–
Non-physical	108	24	.69	.022	.56, .81	.87	<.001	.79, .95	.222
Physical	108	24	.60	.233	.47, .73	.74	.001	.68, .89	–
Any coercive measure	108	20	.59	.305	.45, .74	.84	<.001	.76, .92	–
Medication	108	19	.60	.303	.45, .75	.87	<.001	.75, .98	–
Restraint	108	20	.59	.305	.45, .74	.84	<.001	.74, .98	–
2 years after discharge									
Any violent behavior	107	37	.61	.133	.47, .73	.86	<.001	.80, .93	–
Non-physical	107	35	.60	.163	.49, .72	.79	<.001	.70, .88	–
Physical	107	31	.60	.205	.48, .71	.76	<.001	.66, .86	–
Any coercive measure	107	29	.60	.215	.48, .72	.84	<.001	.76, .91	–
Medication	107	26	.61	.197	.48, .73	.88	<.001	.81, 94	–
Restraint	107	25	.65	.072	.52, .77	.86	<.001	.78, .94	–
Legal outcomes									
Any readmission									
6 months readmission	109	43	.51	.752	.39, .63	.76	<.001	.66, .86	–
1 year readmission	108	51	.52	.680	.41, .63	.77	<.001	.68, .86	–
2 years readmission	107	66	.51	.757	.40, .62	.77	<.001	.68, .86	–
Involuntary readmission									
6 months follow-up	109	15	.61	.268	.42, .80	.71	.031	.55, .87	–
1 year follow-up	108	20	.58	.399	.42, .73	.73	.005	.60, .86	–
2 years follow-up	107	30	.54	.566	.41, .66	.76	<.001	.65, .86	–

BVC, Brøset Violence Checklist; V-RISK-10, Violence Risk Screening-10.

### Differences in predictive validity

The differences in the predictive validities of BVC and V-RISK-10 were analyzed using DeLong tests and are shown in the last column of **Tables 2**, **3**. For the 24-hour period, there were significant differences for every violence criterion (*p* <.013) and any coercive measure (*p* = .019). However, for every single coercive measure 24 hours after admission, there were no differences between the instruments (medication: *p* = .097, restraint: *p* = .214).

Within the 72-hour period after admission, there were significant differences for all violence criteria (all *p* <.019). For coercive measures occurring up to 72 hours after admission, there were significant differences between BVC and V-RISK-10 for any coercive measure and medication (both *p* <.025), but not for restraints (*p* = .083).

For events occurring until discharge, there were no significant differences between either instrument for any criterion (all *p* >.128).

For events after discharge, there was one significant difference between the BVC and V-RISK-10 concerning any violent behavior three months after admission (*p* = .008). For non-physical violence three and twelve months after discharge, there were no differences (both *p* >.222).

### Incremental validity

The incremental predictive validity was examined using stepwise binomial logistic regression with the BVC and V-RISK-10 at admission as predictors and with every outcome as a dependent variable. The analyses revealed an incremental validity exclusively for restraint 24 hours after admission, any violence and non-physical violence occurring until discharge. The results for any violence and non-physical violence in the regression analyses were identical because in this subgroup, every violent act included at least one non-physical violent act (both *n* = 36, see also **Table 2**). Therefore, solely the analysis for any violence will be reported.

For restraint 24 hours after admission, the first step with the BVC revealed a significant regression model, *b(exp)* = .98, *p* <.001, Nagelkerke-*R* ^2^ = .47, *p* <.001. After adding the V-RISK-10 to the model, the LL test revealed a significant change for the overall model fit (χ^2 ^= 5.37, *p* = .020, Nagelkerke-*R* ^2^ = .53, *p* <.001). Both BVC and V-RISK-10 were significant predictors (BVC: *b(exp)* = .77, *p* <.001, V-RISK-10: *b(exp)* = .21, *p* = .030).

BVC as predictor for any violence until discharge revealed a significant regression model (*b(exp)* = .96, *p* <.001, Nagelkerke-*R* ^2^ = .47, *p* <.001). In the second step with the V-RISK-10, the LL test showed a significant change for the overall model fit (χ^2 = ^4.75, *p* = .029, Nagelkerke-*R* ^2^ = .52, *p* <.001). Both BVC and V-RISK-10 significantly contributed to the prediction of any violence until discharge (BVC: *b(exp)* = .80, *p* <.001, V-RISK-10: *b(exp)* = .15, *p* = .031).

### Further results

**Table 4** shows optimal cut-offs, sensitivity, and specificity for the BVC and V-RISK-10 for violence after admission and discharge.

**Table 4:** Optimal cut-off points, sensitivity and specificity for BVC and V-RISK-10.

	BVC^a^			V-RISK-10^b^		
Variables	Cut-off	sensitivity	specificity	Cut-off	sensitivity	specificity
24 hours after admission						
Any violent behavior	3.5	83	90	12.1	92	70
Non-physical	3.5	83	89	12.1	91	70
Physical	3.5	84	99	12.1	96	63
72 hours after admission						
Any violent behavior	2.5	83	84	11.6	83	70
Non-physical	2.5	83	83	10.5	83	69
Physical	2.5	95	79	12.1	85	67
Until discharge						
Any violent behavior	2.5	72	86	12.1	75	78
Non-physical	2.5	72	86	12.1	75	78
Physical	3.5	80	80	12.1	80	71
3 months after discharge						
Any violent behavior	–	–	–	13.9	94	94
Non-physical	–	–	–	13.9	94	87
Physical	–	–	–	14.1	54	89
1 year after discharge						
Any violent behavior	–	–	–	10.5	79	80
Non-physical	–	–	–	10.5	83	79
Physical	–	–	–	10.5	83	79

AUC, Area under the curve; BVC, Brøset Violence Checklist; V-RISK-10, Violence Risk Screening-10.

^a^Cut-off-points were calculated for the BVC sum score at admission.

^b^Cut-off points for post-admission events were derived from V-RISK-10 sum scores at admission, and for post-discharge events, from scores at discharge.

## Discussion

The aim of this study was to examine the predictive validity of a German-translated version of the V-RISK-10 for long-term events. We collected a database of involuntarily admitted patients in a general psychiatric unit and compared the predictive validity between the V-RISK-10 and the BVC (an established risk assessment screening for short-term events). Various outcomes, including violence, coercive measures and readmission were examined. The results replicate the findings of the BVC for imminent violence and associated events. Moreover, our findings support the predictive validity of the V-RISK-10 for long-term events within the context of individuals who were involuntarily admitted. There is some evidence that both instruments together exhibit incremental validity for restraint and violence.

The BVC demonstrated large effects with most of the short-term events up to 72 hours. This result aligns with previous studies (23). Additionally, the BVC was associated with events extending beyond the initial 72-hour period after admission. This observation introduces a novel aspect, as many recent studies focus on a follow-up period between 6-24 hours (23). Considering its highly efficient use within a two-minute period, this finding emphasizes the potential of the BVC to serve as a valuable and economic screening tool for individuals undergoing an involuntary admission.

The V-RISK-10 showed large effects for long-term events occurring until discharge. For long-term events after discharge, solely the V-RISK-10 demonstrated substantial predictive validity compared to the BVC (except for any violence and non-physical violence both 3 and 12 months after discharge). This finding is aligned with the underlying conceptual design of both instruments (7). The BVC was developed to predict events taking place in the following days (15), while the V-RISK-10 was designed to anticipate events spanning the entire duration of stay in the hospital, extending beyond discharge (16, 17). Additionally, the V-RISK-10 showed predictive validities for short-term events. Despite this, predictive validities were comparably weaker than those observed for the BVC.

Our findings support incremental validity of both BVC and V-RISK-10 in predicting restraint 24 hours after admission and violence up until discharge. There is some evidence that combining both instruments might enhance the predictive validity of both criteria. However, it is important to note that the contribution of the V-RISK-10 to the prediction is relatively low to that of the BVC. Using V-RISK-10 and BVC together to predict restraint 24 hours after admission could potentially prompt primary preventive interventions aimed at avoiding such events. The incremental effect for violence, including physical and non-physical violence, could be seen as one indicator for the utilization to a variety of endangerment. This aspect could hold of particular significance in evaluating the criterion of endangerment during the admission process (14).

The results revealed that different diagnostic accuracies were identified. We found higher cut-offs for both BVC and V-RISK-10 compared to previous studies (e.g., 17, 19, 21, 27). This divergence may arise from our focus on involuntarily admitted individuals. As these individuals inherently constitute a high-risk population, there is evidence that prior information might influence diagnostic accuracy (35). For instance, in other areas like suicidality, the diagnostic accuracy of structured methods appears to depend on prior information, such as previous suicide attempts (36). This leads to the conclusion that cut-off points derived from other studies of the BVC and V-RISK-10 may not be directly applicable to high-risk populations. Instead, these thresholds should be recalibrated specifically for involuntarily admitted individuals.

Altogether, the observations suggest that the BVC and the V-RISK-10 have the potential to distinguish between individuals at high and low risk within a high-risk population of individuals involuntarily admitted. Additionally, it gives further insight into criteria that are critical to the treatment process of this specific population, such as coercive measures and violence. Given the ethical challenges of involuntary admissions, the findings highlight that both instruments might facilitate more objective evaluations, mitigating potential bias stemming from subjective impressions, time-consuming procedures, and pressure by third parties during the admission process (37, 38). Because both instruments do not require a specific training, they could support the evaluation process during an involuntary admission, possibly even for professionals without medical expertise (39). In summary, this might help avoid unnecessary and burdensome involuntary stays.

Nevertheless, as involuntary admission can also function as primary preventive interventions for individuals who did not exhibit any signs of endangerment prior to admission, standardized procedures present specific disadvantages and pitfalls. First, the absence of individualization and specific context information in structured risk-assessment instruments may neglect factors such as physical disabilities and advanced age, contributing to false decisions. Moreover, based on prior knowledge by responsible healthcare professionals, “high-risk” individuals (according to risk assessment) could sometimes profit from alternative interventions (37, 40, 41). Second, though standardized screening procedures improve the hit rate, for some individuals it might lead to unfair results. When it comes to predicting violent behavior after admission in our sample, V-RISK-10 would have falsely identified 22-37% of the individuals as potentially violent, even though they did not exhibit any violent behavior. Third, some risk assessments lack flexibility. This is particularly applicable for the V-RISK-10, in which half of the items include static factors such as previous and current psychotic features, violence, or substance abuse. These concerns are particularly pertinent as many cases involve primary preventive involuntary admission.

There are several limitations in our study. The first limitation reflects the retrospective design that results in several consequences based on an observer (e.g. interpretation of violence) and confirmation bias (coding of the BVC and V-RISK-10 by first author, T.K.). For short-term events within 24 hours, information was not always precise enough to differentiate whether behavioral aspects were antecedents or accompanying symptoms of the events (see **Table 3** concerning the sensitivity for BVC and physical violence). For this reason, we decided to add a longer period of 72 hours to avoid circular reasoning. Because the dataset was originally not collected for scientific purpose, we assume a lack of information for clinical data, such as psychiatric history, personality styles and/or disorders, staging and rebound-phenomena after patients stop taking their medication: information that is essential for the V-RISK-10 with historical items. Furthermore, because the study relies on patients who have been involuntarily admitted to a psychiatric ward, conclusions for other patients in general psychiatric wards and settings are limited. Because this population comprises high risk constellative factors, a study with patients who have other risk profiles might produce accordingly higher variance with more robust results.

Despite the limitations, this study is, to the best of our knowledge, the first study that examines the predictive validity of a German-translated version of the V-RISK-10. It shows predictive validity for both short- and long-term events after admission and discharge. As an ARAI, it could be an effective option for medical staff in general psychiatric hospitals and employees of the SPS who are related to the admission process. As expected, the BVC shows predictive validity for short-term events and no predictive validity for long-term events after discharge. It shows further predictive validity for events beyond the 72 hour period. Altogether, the results support the idea that the V-RISK-10 could be superior in the long-term prognosis. However, the results for the incremental validity showed that simultaneous application does lead to an added value, although it is relatively small. Cut-offs for both instruments were higher compared to previous studies because we examined a high-risk population. For future research, prospective designs with larger and more diverse samples are needed. Moreover, since endangerment towards others is one factor that contributes to involuntary admissions, further studies could extent their scope to examine additional conditions reflecting self-endangerment, including but not limited to suicidality and delirant symptoms.

## Data availability statement

The original contributions presented in the study are included in the article/supplementary materials, further inquiries can be directed to the corresponding author/s.

## Ethics statement

The studies involving humans were approved by Charité-Universitätsmedizin Berlin (EA4/006/23). The studies were conducted in accordance with the local legislation and institutional requirements. Written informed consent for participation was not required from the participants or the participants’ legal guardians/next of kin in accordance with the national legislation and institutional requirements.

## Author contributions

TK: Conceptualization, Formal analysis, Investigation, Methodology, Writing – original draft. PB: Project administration, Supervision, Writing – review & editing. JH: Supervision, Writing – review & editing.

## References

[1] Wertz M Schobel S Schiltz K Rettenberger M. A comparison of the predictive accuracy of structured and unstructured risk assessment methods for the prediction of recidivism in individuals convicted of sexual and violent offense. Psychol Assess. (2022) 35:152–64. doi: 10.1037/pas0001192 36455027

[2] Grove WM Zald DH Lebow BS Snitz BE Nelson C. Clinical versus mechanical prediction: a meta-analysis. Psychol Assess. (2000) 12:19–30. doi: 10.1037/1040-3590.12.1.19 10752360

[3] Falzer PR. Valuing structured professional judgment: Predictive validity, decision-making, and the clinical-actuarial conflict. Behav Sci Law. (2013) 31:40–54. doi: 10.1002/bsl.2043 23339121

[4] Ægisdóttir S White MJ Spengler PM Maugherman AS Anderson LA Cook RS. The meta-analysis of clinical judgment project: Fifty-six years of accumulated research on clinical versus statistical prediction. Couns Psychol. (2006) 34:341–82. doi: 10.1177/0011000005285875

[5] Ogonah MG Seyedsalehi A Whiting D Fazel S. Violence risk assessment instruments in forensic psychiatric populations: A systematic review and meta-analysis. Lancet Psychiatry. (2023) 10:780–9. doi: 10.1016/S2215-0366(23)00256-0 PMC10914679 37739584

[6] Singh JP Desmarais SL Van Dorn RA. Measurement of predictive validity in violence risk assessment studies: A second-order systematic review. Behav Sci Law. (2013) 31:55–73. doi: 10.1002/bsl.2053 23444299

[7] Anderson KK Jenson CE. Violence risk–assessment screening tools for acute care mental health settings: Literature review. Arch Psychiatr Nurs. (2019) 33:112–9. doi: 10.1016/j.apnu.2018.08.012 30663614

[8] Iozzino L Ferrari C Large M Nielssen O De Girolamo G. Prevalence and risk factors of violence by psychiatric acute inpatients: a systematic review and meta-analysis. PloS One. (2015) 10:e0128536. doi: 10.1371/journal.pone.0128536 26061796 PMC4464653

[9] McNiel DE Binder RL. Predictive validity of judgments of dangerousness in emergency civil commitment. Am J Psychiatry. (1987) 144:197–200. doi: 10.1176/ajp.144.2.197 3812787

[10] Akther SF Molyneaux E Stuart R Johnson S Simpson A Oram S. Patients' experiences of assessment and detention under mental health legislation: Systematic review and qualitative meta-synthesis. BJPsych Open. (2019) 5:e37. doi: 10.1192/bjo.2019.19 31530313 PMC6520528

[11] Katsakou C Priebe S. Outcomes of involuntary hospital admission–a review. Acta Psychiatr Scand. (2006) 114:232–41. doi: 10.1111/j.1600-0447.2006.00823.x 16968360

[12] Flemmerer M Bühling-Schindowski F Baumgardt J Bechdolf A. Predictors of the use of restraint in inpatient psychiatric care among patients admitted via the emergency department. J Psychiatr Res. (2023) 162:37–43. doi: 10.1016/j.jpsychires.2023.03.043 37086605

[13] Müller M Brackmann N Jäger M Theodoridou A Vetter S Seifritz E. Predicting coercion during the course of psychiatric hospitalizations. Eur Psychiatry. (2023) 1-27. doi: 10.1192/j.eurpsy.2023.3 PMC9981454 36700423

[14] Marschner R Lesting W Stahmann R. Freiheitsentziehung und Unterbringung. München: Beck (2019).

[15] Almvik R Woods P Rasmussen K. The Brøset Violence Checklist: Sensitivity, specificity, and interrater reliability. J Interpers Violence. (2000) 15:1284–96. doi: 10.1177/088626000015012003

[16] Hartvig P Roaldset JO Moger TA Østberg B Bjørkly S. The first step in the validation of a new screen for violence risk in acute psychiatry: The inpatient context. Eur Psychiatry. (2011) 26:92–9. doi: 10.1016/j.eurpsy.2010.01.003 20456927

[17] Roaldset JO Hartvig P Bjørkly S. V-RISK-10: Validation of a screen for risk of violence after discharge from acute psychiatry. Eur Psychiatry. (2011) 26:85–91. doi: 10.1016/j.eurpsy.2010.04.002 20619612

[18] Mostafavian Z Hosseini G Masoudi E. Validity and reliability of the Persian version of Violence Risk Screening-10 Instrument (V-Risk-10) in admitted patients to the psychiatric ward. J Res Med Sci. (2022) 27:51. doi: 10.4103/jrms.JRMS_359_19 36092486 PMC9450254

[19] Yao X Li Z Arthur D Hu L Cheng G. Validation of the Violence Risk Screening-10 instrument among clients discharged from a psychiatric hospital in Beijing. Int J Ment Health Nurs. (2014) 23:79–87. doi: 10.1111/j.1447-0349.2012.00890.x 23360576

[20] Lockertsen Ø Varvin S Færden A Eriksen BMS Roaldset JO Procter NG. Risk assessment of imminent violence in acute psychiatry: a step towards an extended model. J Forens Psychiatry Psychol. (2020) 31:41–63. doi: 10.1080/14789949.2019.1663898

[21] Roaldset JO Gustavsen CC Lockertsen Ø Landheim T Bjørkly SK. Validation of a violence risk screening for youth in psychiatric inpatient care—a pilot study of V-RISK-Y. Front Psychiatry. (2023) 14:1210871. doi: 10.3389/fpsyt.2023.1210871 37614654 PMC10443591

[22] Linaker OM Busch-Iversen H. Predictors of imminent violence in psychiatric inpatients. Acta Psychiatr Scand. (1995) 92:250–4. doi: 10.1111/j.1600-0447.1995.tb09578.x 8848948

[23] Hvidhjelm J Berring LL Whittington R Woods P Bak J Almvik R. Short-term risk assessment in the long term: A scoping review and meta-analysis of the Brøset Violence Checklist. J Psychiatr Ment Health Nurs. (2023) 30(4):637–48. doi: 10.1111/jpm.12905 36718598

[24] Abderhalden C Needham I Miserez B Almvik R Dassen T Haug HJ. Predicting inpatient violence in acute psychiatric wards using the Brøset-Violence-Checklist: A multicentre prospective cohort study. J Psychiatr Ment Health Nurs. (2004) 11:422–7. doi: 10.1111/j.1365-2850.2004.00733.x 15255916

[25] Bjørkly S Hartvig P Heggen F-A Brauer H Moger T. Development of a brief screen for violence risk (V-RISK-10) in acute and general psychiatry: An introduction with emphasis on findings from a naturalistic test of interrater reliability. Eur Psychiatry. (2009) 24:388–94. doi: 10.1016/j.eurpsy.2009.07.004 19716682

[26] World Health Organization. Process of translation and adaptation of instruments (2016). Available online at: http://www.who.int/substance_abuse/research_tools/translation/en/.

[27] Yao X Li Z Arthur D Hu L Cheng G. The application of a violence risk assessment tool among Chinese psychiatric service users: a preliminary study. J Psychiatr Ment Health Nurs. (2012) 19:438–45. doi: 10.1111/j.1365-2850.2011.01821.x 22073978

[28] Monahan J Steadman HJ Robbins PC Appelbaum P Banks S Grisso T. An actuarial model of violence risk assessment for persons with mental disorders. Psychiatr Serv. (2005) 56:810–5. doi: 10.1176/appi.ps.56.7.810 16020812

[29] Cantor SB Kattan MW. Determining the area under the ROC curve for a binary diagnostic test. Med Decis Making. (2000) 20:468–70. doi: 10.1177/0272989X0002000410 11059479

[30] DeLong ER DeLong DM Clarke-Pearson DL. Comparing the areas under two or more correlated receiver operating characteristic curves: a nonparametric approach. Biometrics. (1988) 44(3): 837–45. doi: 10.2307/2531595 3203132

[31] Rice ME Harris GT. Comparing effect sizes in follow-up studies: ROC Area, Cohen's d, and r. Law Hum Behav. (2005) 29:615–20. doi: 10.1007/s10979-005-6832-7 16254746

[32] Youden WJ. Index for rating diagnostic tests. Cancer. (1950) 3:32–5. 10.1002/1097-0142(1950)3:1<32::aid-cncr2820030106>3.0.co;2-3 15405679

[33] Rudolf M Buse J. Multivariate Verfahren: Eine praxisorientierte Einführung mit Anwendungsbeispielen. Göttingen: Hogrefe (2020).

[34] RStudio Team. RStudio: Integrated Development for R. RStudio. Boston, MA: PBC (2020).

[35] Deeks JJ Altman DG. Diagnostic tests 4: likelihood ratios. BMJ. (2004) 329:168–9. doi: 10.1136/bmj.329.7458.168 PMC478236 15258077

[36] Riblet NB Matsunaga S Lee Y Young-Xu Y Shiner B Schnurr PP. Tools to detect risk of death by suicide: A systematic review and meta-analysis. J Clin Psychiatry. (2022) 84:43891. doi: 10.4088/JCP.21r14385 PMC9890591 36383739

[37] Marty S Jaeger M Moetteli S Theodoridou A Seifritz E Hotzy F. Characteristics of psychiatric emergency situations and the decision-making process leading to involuntary admission. Front Psychiatry. (2019) 9:760. doi: 10.3389/fpsyt.2018.00760 30713511 PMC6345710

[38] Røtvold K Wynn R. Involuntary psychiatric admission: Characteristics of the referring doctors and the doctors’ experiences of being pressured. Nord J Psychiatry. (2015) 69:373–9. doi: 10.3109/08039488.2014.987165 25536143

[39] Roaldset JO Hartvig P Bjørkly S. Psychometric properties and predictive validity of a police version of a violence risk screen–A pilot study. Int J Law Psychiatry. (2017) 54:133–9. doi: 10.1016/j.ijlp.2017.06.007 28668227

[40] Henderson C Flood C Leese M Thornicroft G Sutherby K Szmukler G. Effect of joint crisis plans on use of compulsory treatment in psychiatry: single blind randomised controlled trial. Bmj. (2004) 329:136. doi: 10.1136/bmj.38155.585046.63 15240438 PMC478218

[41] Khazaal Y Chatton A Pasandin N Zullino D Preisig M. Advance directives based on cognitive therapy: A way to overcome coercion related problems. Patient Educ Couns. (2009) 74:35–8. doi: 10.1016/j.pec.2008.08.006 18829211

